# Bacteriocin production: a relatively unharnessed probiotic trait?

**DOI:** 10.12688/f1000research.9615.1

**Published:** 2016-10-27

**Authors:** James W. Hegarty, Caitriona M. Guinane, R. Paul Ross, Colin Hill, Paul D. Cotter

**Affiliations:** 1 Teagasc Food Research Centre, Moorepark, Fermoy, Co. Cork, Ireland; 2 Department of Microbiology, University College Cork, Cork, Ireland; 3 APC Microbiome Institute, University College Cork, Cork, Ireland

**Keywords:** probiotic, bacteriocin, microbiota, gut health

## Abstract

Probiotics are “live microorganisms which, when consumed in adequate amounts, confer a health benefit to the host”. A number of attributes are highly sought after among these microorganisms, including immunomodulation, epithelial barrier maintenance, competitive exclusion, production of short-chain fatty acids, and bile salt metabolism. Bacteriocin production is also generally regarded as a probiotic trait, but it can be argued that, in contrast to other traits, it is often considered a feature that is desirable, rather than a key probiotic trait. As such, the true potential of these antimicrobials has yet to be realised.

## What are bacteriocins?

Bacteriocins are small, heat-stable, ribosomally synthesised antimicrobial peptides produced by bacteria that are active against other bacteria and to which the producer is immune ^1^. These peptides exhibit considerable diversity with respect to their size, structure, mechanism of action, inhibitory spectrum, immunity mechanisms, and target cell receptors ^2^. Indeed, for example, many bacteriocins have a narrow spectrum of activity, displaying antimicrobial activity against strains that are closely related to the producer, whereas others display antimicrobial activity across a broad variety of different genera ^1^. The regulation of bacteriocin production can be complex, in some instances being influenced by environmental conditions such as pH, temperature, and growth medium ^3– 5^.

Despite the diversity among bacteriocins, they can generally be classified into one of two groups on the basis of whether they undergo post-translational modifications ^1^. Class I (modified) bacteriocins have been further subdivided into the following subgroups: lantibiotics, linaridins, linear azol(in)e-containing peptides, cyanobactins, thiopeptides, lasso peptides, sactibiotics, glycocins, and modified microcins ^6^. Class II (unmodified) bacteriocins consist of five subgroups: four correspond to the unmodified lactic acid bacteria (LAB) bacteriocins and one corresponds to the unmodified microcins and includes class IIa (pediocin-like), IIb (two-peptide bacteriocins), IIc (circular bacteriocins), IId (linear, non-pediocin-like bacteriocins), and IIe (microcin E492-like bacteriocins).

Antimicrobial/bacteriocin production may contribute to probiotic functionality through three different mechanisms ^7^: firstly, as colonising peptides, bacteriocins aid the survival of the producing strain in the gut environment ^8^; secondly, bacteriocins function through direct inhibition of the growth of pathogens ^9^; and, finally, bacteriocins may serve as signalling peptides/quorum-sensing molecules in the intestinal environment ^10^. However, although bacteriocin production is generally regarded as a probiotic trait, it can be argued that, in contrast to other traits, it is often considered a feature that is desirable, rather than a key probiotic trait. As such, the true potential of these peptides for gut health, and indeed other applications ^11^, has yet to be realised.

## Bacteriocin-producing probiotic strains

**Figure 1.**
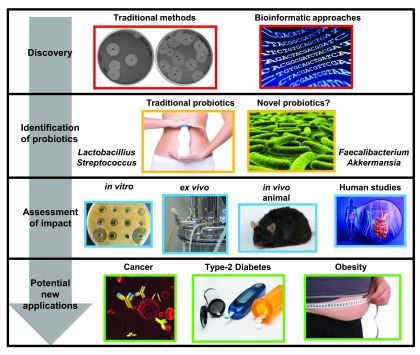
Bacteriocins, from discovery to potential probiotic application. Strategies to identify new bacteriocins include culture-based methods and newer bioinformatics-based approaches. These can lead to the identification of bacteriocin-producing strains from traditionally utilised, or novel, probiotic species. The impact of a bacteriocin-producing strain on health can be assessed using *in vitro*, *ex vivo*, and *in vivo* methods and, depending on the outcome, has the potential to be applied to prevent or treat various disease states.

Probiotics are “live microorganisms which, when consumed in adequate amounts, confer a health benefit to the host” ^12^. The majority of probiotic species in commercial use today are representatives of the genera *Lactobacillus* or *Bifidobacterium*. However, despite the health-promoting attributes associated with *Bifidobacterium* spp. and their potential ability to produce these antimicrobials, there is limited information available regarding functional bacteriocin production by bifidobacteria ^13^. This raises the following question: is bacteriocin production a rare trait among bifidobacteria or are bacteriocin-producing bifodobacteria being overlooked or not being effectively harnessed? Interestingly, while examining the diversity and distribution of bacteriocins from different body sites, Zheng *et al*. reported the absence of bacteriocins produced by *Bifidobacterium* spp. in the gut, despite bifidobacteria accounting for up to 10% of the microbiome ^14^. Walsh *et al*. identified just two novel putative bacteriocin gene clusters, belonging to the lantibiotic class, from two *Bifidobacterium* spp. during a screen of the gastrointestinal (GI) tract subset of the Human Microbiome Project reference genome database ^15^, again emphasising the rarity of production among this genus. Other probiotics include specific strains of *Streptococcus* spp., *Lactococcus* spp., and *Enterococcus* spp. as well as the *Escherichia coli* strain Nissle 1917 and yeasts such as *Saccharomyces boulardii* ^16, 17^. As lactococci are not typically regarded as gut-associated microorganisms and the use of enterococci as probiotics is controversial, for the purposes of this review we have focused on reviewing what is known about bacteriocin production from among probiotic lactobacilli and streptococci of human origin and discussing the extent to which this trait is valued when commercialising associated strains ( Figure 1).

### Probiotic lactobacilli

The mechanism by which bacteriocin production contributes to probiotic functionality among species of *Lactobacillus* has been the focus of a number of studies. van Hemert *et al*. reported that genes required for plantaricin production and transport contributed to the immunomodulatory effects of *Lactobacillus plantarum* WCFS1 on peripherial blood mononuclear cells ^10^. Using the same strain of *L*. *plantarum*, Meijerink *et al*. established that six of the eight genes that modulate the dendritic cell cytokine response were involved in bacteriocin production or secretion ^18^. The beneficial impact of using *L*. *johnsonii* La1 to control *Helicobacter pylori* colonisation was also previously examined ^19^.

A number of strains of *Lactobacillus salivarius* which possess probiotic traits have been identified, and the genus is also associated with the production of a number of class II (a, b, and d) bacteriocins ^20– 24^. Despite the fact that bacteriocins produced by potential probiotic strains have significant promise as alternative treatments to target clinically relevant pathogens, the degree to which they are expressed under the harsh conditions within the GI tract has not been studied in great detail. For the same reason, strategies have not been developed to ensure that bacteriocin production is triggered within this environment. It has only been established that certain bacteriocins produced by *L*. *salivarius* strains can indeed be produced within many of the stressful conditions encountered in the gut ^3, 25^. *L*. *salivarius* UCC118 (NCIMB 40829 LSUCC118) is a very well-characterised strain that has been studied with a view to potential probiotic applications and that notably produces the class II, two-peptide bacteriocin Abp118 ^26^. Abp118 displays a relatively broad spectrum of antimicrobial activity against a number of food-borne and medically significant pathogens ^27^. This probiotic strain was the focus of particular attention when it was employed in an important ‘proof-of-concept’ study, which proved that bacteriocin production is indeed a probiotic trait by virtue of its ability to protect mice against *Listeria monocytogenes* infection ^9^. The strain and a non-bacteriocin-producing equivalent were also used by Murphy *et al*. to test their relative abilities to mitigate the metabolic abnormalities associated with obesity in a diet-induced obesity (DIO) mouse model and modulate the gut microbiota as a potential driver of these abnormalities ^28^. Although reductions in weight gain were evident among animals that received the bacteriocin-producing strain, these effects were transient. It was notable that the composition of the murine gut microbiota differed depending on which strain they were fed, indicating *in situ* functionality ^28^. As the ability to adhere to intestinal epithelium can play a role in probiotic functionality, this strain has also been examined to assess the influence of adhesion to intestinal epithelial cells on gene expression. Notably, bacteriocin gene expression was induced upon adhesion to epithelial cells, possibly through a mechanism whereby the presence of an induction peptide at a high enough local concentration triggers bacteriocin production. The phenomenon was observed for the UCC118 wild-type strain but not an *srtA* mutant, as disruption of the sortase gene *srtA* results in significantly lower levels of adhesion ^29^, following exposure to Caco-2 cells ^30^. It is notable that, despite UCC118 being perhaps the probiotic strain in which the benefits of bacteriocin production are clearest, the strain has yet to be brought to market. Another *L*. *salivarius*-produced bacteriocin that has been the focus of investigation is bactofencin A, a class IId bacteriocin ^24^. This bacteriocin is unusual in that it does not share significant homology with previously characterised bacteriocins but instead is more similar to a group of eukaryotic antimicrobial peptides ^24^. Bactofencin A has a relatively broad spectrum of activity, inhibiting two clinically significant pathogens: *Staphylococcus aureus* and *L*. *monocytogenes* ^24^. The impact of the bactofencin A-producing strain on intestinal populations and microbial diversity in a simulated model of the distal colon has been examined ^25^ and was found to alter the proportions of a number of important gut genera, including *Fusobacterium*, *Bacteroides*, and *Bifidobacterium*, resulting in a positive, albeit subtle, effect on gut populations ^25^.

Despite the research described above, bacteriocin production among commercial probiotic lactobacilli has, in general, not been studied in great detail, and the information available regarding which commercial probiotics produce bacteriocins and which bacteriocins are produced most frequently is limited. *Lactobacillus acidophilus* probiotics, several of which are employed for use in commercial products ^31, 32^, are somewhat exceptional in this regard in that two such strains, NCFM and LA-5, are known to produce the bacteriocin lactacin B ^33, 34^. This bacteriocin has a narrow spectrum of activity, capable of inhibiting other lactobacilli and *Enterococcus faecalis* ^35^. Notably, with respect to this commentary, the contribution of lactacin B, if any, to probiotic functionality has not been determined.

### *Streptococcus salivarius* 

*Streptococcus salivarius* is a well-characterised human commensal of the oral cavity ^36^ and has been found to colonise within just a few hours of birth ^37^. It is also a common inhabitant of the gut, particularly the stomach and jejunum. Some strains of *S*. *salivarius* have gained attention because of their role as safe and effective probiotics, and have been employed to promote a healthy oral microbiota ^38, 39^. As reviewed by Wescombe *et al*., strain K12 is the model *S*. *salivarius* probiotic and is available in commercial preparations (BLIS K12; BLIS Technologies, Otago, New Zealand). K12 was initially selected because of its ability to inhibit the pathogen *Streptococcus pyogenes*, but now several other health-promoting effects have been noted ^40^. This includes the ability to inhibit group B streptococci (GBS) ^41^, including isolates suspected of causing disease in newborns and colonising isolates from the vaginal tract of pregnant women. Some of these activities were dependent, or partially dependent, on the presence of a megaplasmid that encodes the salivaricin A2 and salivaricin B bacteriocins ^41^.

Other strains of *S*. *salivarius* examined for their probiotic application include M18, which contains a megaplasmid encoding a number of bacteriocins ^42^. To evaluate its probiotic potential, the impact of this strain to prevent or reduce the risk of dental caries and influence dental health was examined in a randomised, double-blind, placebo-controlled trial ^43^. The persistence of this strain in saliva was also investigated and revealed to be dose dependent ^44^. This study demonstrated *in vitro* transfer of the bacteriocin-encoding megaplasmids between two strains of *S*. *salivarius*. This may allow the enhancement of probiotic strains by transferring the megaplasmid from those that persist poorly but demonstrate strong bacteriocin production to indigenous *S*. *salivarius* that persist strongly but demonstrate poor bacteriocin production ^44^. Additionally, the identification of novel bacteriocins, including salivaricin 9 ^45^ and the recently identified salivaricin E ^46^, from this species continues to enhance the probiotic potential of *S*. *salivarius*.

## Novel health targets for bacteriocins

The ability of bacteriocins to modulate the gut microbiota by targeting undesirable components without having a negative impact on the beneficial populations is an attractive trait. The role by which a bacteriocin could regulate niche competition among enterococci or between enterococci and the intestinal microbiota was examined by Kommineni *et al*. ^47^. Here, it was demonstrated that *E*. *faecalis* containing the conjugative pPD1 plasmid, which expresses bacteriocin 21, both replaced indigenous enterococci and outcompeted *E*. *faecalis*, which lacked the plasmid, while the transfer of this plasmid to other *E*. *faecalis* strains enhanced their survival in the intestine. Finally, vancomycin-resistant enterococci were cleared following subsequent colonisation with *E*. *faecalis* harbouring a conjugation-defective pPD1 mutant ^47^. These results do indeed demonstrate that bacteriocin production by commensal bacteria contributes to niche competition and an alternative therapeutic approach to eliminating intestinal colonisation by multidrug-resistant bacteria may be provided by bacteriocins delivered by commensals ^47^.

Janek *et al*. observed a high frequency of bacteriocin production among nasal *Staphylococcus* strains with highly variable antimicrobial activity against other nasal members, suggesting a need to inhibit different competitors ^48^. The diverse activity spectra of bacteriocins within the nose may facilitate the ability of a bacterial species to dominate the resident populations, suggesting the development of probiotics that could promote a desirable microbiota composition and eliminate pathogens such as *S*. *aureus* ^48^.

The majority of studies to date, focus on bacteriocin-producing probiotics that can inhibit well-established gut pathogens. Next-generation sequencing technologies continue to provide a more thorough understanding of the role of the gut microbiota in GI health and, as a result, new targets are emerging. The use of a targeted approach can help to provide further insights into such studies by establishing whether increases in specific taxa are the cause, or a consequence, of such diseases. More specifically, in instances where the link between the putative pathogen and disease is not clear, the targeted removal of the microbe by bacteriocin-based approaches can establish aetiology. Even more significantly, if the target microbe is established to be a pathogen, the bacteriocin can also be employed to prevent/treat disease. Although, yet again, the harnessing of bacteriocin-producing strains to this end has remained a focus of academic research only, here we provide some examples of ways in which these bacteria could be applied.

### Metabolic health

Obesity is a complex syndrome and has a number of serious implications for human health, including cardiovascular disease, type 2 diabetes (T2D), and musculoskeletal disorders. The role of the gut microbiota in obesity and overall metabolic health has received considerable attention in recent years. Initially, it was noted that the gut microbiota of genetically obese mice have been associated with an increase in the phylum Firmicutes and a decrease in the phylum Bacteroidetes ^49, 50^. However, there is conflicting evidence in human studies with regard to what the key populations involved are ^51^. Nonetheless, the ability of the previously mentioned *L*. *salivarius* UCC118 strain to inhibit a number of Firmicutes was part of the logic behind investigating its ability to control weight gain in DIO mice ^28^. More recent research has specifically highlighted populations that may play a role in obesity or in T2D ^52– 57^ that could be directly or indirectly targeted by antimicrobial action to improve intestinal balance and in turn GI health.

There have been other studies that have more specifically established the role of a particular species or strain in obesity and T2D. Fei and Zhao demonstrated the role of the endotoxin-producing *Enterobacter cloacae* B29 in inducing obesity and insulin resistance in germfree mice ^58^. It was also shown that *Clostridium ramosum*, a species previously shown to be enriched in patients with T2D ^56^, promoted obesity in a gnotobiotic mouse model fed a high-fat diet ^59^. Bacteriocins produced within the gut with specific activity against some of these organisms may be effective in beneficially balancing metabolic health.

### Cancer

There have been some suggestions that bacteriocins can be employed as anticancer agents, either through their impact on cancerous cells or through the inhibition of bacteria associated with the initiation of disease ^60^. One such study focused on the impact of nisin on head and neck squamous cell carcinoma (HNSCC) cell apoptosis and cell proliferation *in vitro* and *in vivo* in murine oral cancer ^61^. It was revealed that treatment with increasing concentrations of nisin induced increasing DNA fragmentation and apoptosis on three different cancer cell lines. In the oral cancer mouse model, groups receiving nisin showed reduced tumour volumes through activation of CHAC1 expression when compared with controls, while pre-treating with nisin prior to and three weeks after tumour cell inoculation led to the same effect ^61^. It was suggested that in this study the selective action of nisin arose from structural differences in the composition of the plasma membranes between HNSCC cells and primary keratinocytes. Although it was the nisin peptide rather than the bacteriocin-producing strain that was used, it would be interesting if strains capable of producing nisin or its variants could be used in a similar manner.

In the context of inhibiting potentially cancer-causing microbes, we refer to the example of *Fusobacterium nucleatum* ^62^. Though initially regarded as a component of the oral cavity, *F*. *nucleatum* is also present in the gut and has been linked to playing a part in different GI disorders such as colorectal cancer (CRC), inflammatory bowel disease, and appendicitis ^63– 66^. The mechanism by ** which *F*. *nucleatum* is thought to promote CRC has been investigated ^67, 68^. As members of the genus *Fusobacterium*, and in particular *F*. *nucleatum*, play a role in numerous disease states as mentioned above, they represent an ideal target for bacteriocin-producing probiotics, but, yet again, this potential has yet to be harnessed.

## Future perspectives

This review highlights the potential for bacteriocins and bacteriocin-producing probiotics as novel therapeutic treatments in many disease states, including the targeting of newly emerging pathobionts involved in a variety of gut disorders. While there is an abundance of knowledge on the application of bacteriocin-producing strains with probiotic potential in an *in vitro* setting, less is known of their impact in an *in vivo* environment and even less again with regard to their application in human health. This is undoubtedly the primary hurdle that needs to be overcome in order for the potential of the multitude of bacteriocin-producing strains that continue to be identified using traditional methods ^69– 71^ or bioinformatic approaches ^14, 15^ to be realised.

In addition to identifying new targets, recent studies have identified *Akkermansia muciniphila* ^72^ and *Faecalibacterium prausnitzii* ^73^ that correlate positively with gut health, as well as a decline in butyrate-producing *Roseburia* species in certain disease states ^56, 57^, which may play a role in future probiotic applications alongside the more traditional strains currently employed. The capacity to produce a bacteriocin by such microbes was demonstrated by Hatziioanou *et al*., who highlighted the first example of a bacteriocin-like substance produced by *Roseburia faecis* M72/1 ^74^. Additionally, *in silico* screens may prove useful in identifying putative bacteriocin gene clusters from these genera/species, such as the sactipeptide-like cluster from *Roseburia intestinalis* L1-82 ^15^. It will be necessary to determine whether these potential probiotics of the future have the ability to produce bacteriocins that can contribute to human health and whether this potential can be more effectively harnessed than has been the case to date. Until such time as this occurs, bacteriocin production will continue to be regarded as a probiotic trait in theory rather than in commercial reality.
